# Hirschsprung’s Disease in Newborns

**Published:** 2013-01-01

**Authors:** Sushmita N Bhatnagar

**Affiliations:** HOD, Pediatric Surgery, B.J.Wadia Hospital for Children, Parel, Mumbai, India.

 (This section is meant for residents to check their understanding regarding a particular topic)

## Questions

1. What are the characteristic presenting features in neonates with Hirschsprung’s disease (HD)?
2. What are the types of Hirschsprung’s disease?
3. What is the initial treatment?
4. How is Hirschsprung’s disease diagnosed?
5. What are the surgical options?
6. What are the long-term sequlae?
7. What are the associated anomalies?

## Answers

1. The presenting features of HD vary depending on the length of the aganglionic segment and the severity. The gestational age at delivery also influences the clinical features, as HD rarely presents in preterm babies. The several methods in which neonatal HD present are:
  **
1. Delayed passage of meconium: **
In healthy term neonates, delay of more than 48 hours is indicative of HD though this feature occurs in only about 50% of neonates with HD.
In preterm babies, delayed passage of meconium is normally seen hence this feature cannot suggest HD. The passage of meconium in newborns should not be initiated by suppository, thermometer or by digital rectal exam, and a careful history is mandatory to establish such intervention. HD cannot be ruled out in babies with such intervention.
**2. Neonatal bowel obstruction: **
Distal bowel obstruction is a presenting feature in about 25% of newborns who have HD and in 15-20% of all intestinal obstructions in the neonates. (1) The classical features are distended abdomen, bilious vomiting, fever, dehydration, lethargy, not passed meconium, and occasionally dilated peristaltic loops visible on the per abdominal examination in the neonate with a normal anus are almost diagnostic of HD. Insertion of simple rubber catheter and baby passing large quantity of meconium and flatus are characteristic of HD.
**3. Neonatal bowel perforation:**
5 percent of children with Hirschsprung’s disease have bowel perforation, colonic (2) or ileal and accounts for about 10 percent of all newborn bowel perforations. Features of perforative peritonitis predominate and in this situation the diagnosis of HD is not clear. Hence, during exploration of such neonates, the colon must be examined and appropriate seromuscular biopsies must be done so as not to miss the diagnosis of HD.
**4. Neonatal enterocoliis:**
Sudden onset of diarrhea with or without blood in the stools in neonates should raise the suspicion of HD. About 30% of neonates with HD develop enterocolitis. The stool is very foul smelling, explosive and is associated with severe abdominal distension, fever, lethargy, dehydration and occasionally generalized sepsis. Hirschsprung’s enterocolitis is a life threatening condition if toxic megacolon develops and identification and treatment must be prompt.
**5. Constipation:**
Constipation is the neonates could be associated with poor feeding, abdominal distension, straining to defecate, failure to thrive, jaundice, occasional non-bilious vomiting and an empty rectum on per rectal examination followed by explosive passage of feces and flatus after removal of the finger. Neonates could be passing normal stools before presenting with constipation. Appropriate investigations should be done if these features are present so as not to misdiagnose the condition.
2. The length of the aganglionic segment anatomically divides HD into 4 types -
  **Short segment HD: ~ 75%: **
  - rectal and distal sigmoid colonic involvement only
  **Long segment HD: ~ 15%: **
  - typically extends to splenic flexure / transverse colon
  **Total colonic aganglionosis: 2 - 13%:**
  - also known as Zuezler – Wilson syndrome
  - occasional extension of aganglionosis into small bowel
  **Ultra-short segment HD:**
  - 3- 4 cm of internal anal sphincter only
  - controversial entity
3. The treatment of a neonate suspected to have HD is dependent on the type of presentation.
**a. Intestinal obstruction:** baby presenting with intestinal obstruction can be treated conservatively if the bowel decompression is possible with high bowel wash with sterile normal saline (water should not be used as large surface of dilated segment of the colon causes water intoxication). In case if bowel washes do not succeed in relieving the obstruction, the baby has to be surgically treated.
The following preliminary steps need to be taken before giving bowel washes
  - Nil by mouth
  - Nasogastric tube insertion
  - Intravenous broad spectrum antibiotics
  - Intravenous fluids – maintenance and correction of dehydration
  - Routine blood investigations
  - Correction of acid-base and electrolyte imbalance if any
  - Stabilization of the child
**b. Intestinal perforation:** Irrespective of the site of perforation and the cause of perforation, the neonate has to be surgically managed after following all the preliminary steps as mentioned above. With a suspicion of HD, the following steps need to be taken intraoperatively -
  - The bowel distal to the perforation has to be examined in great detail for the presence of HD, and the presence of transition zone must be noted and evaluated.
  - Formation of stoma has to be done based on clinical decision and intra-operative findings. Frozen section facilities even if available may not provide an accurate diagnosis of HD in the event of perforative peritonitis and serositis of the bowel wall.
  - In the absence of a transition zone, multiple seromuscular biopsies must be done to rule out HD and if aganglionosis is found on histopathology, definitive surgical intervention must be undertaken.
  **c. Enterocolitis: **Neonate presenting with enterocolitis is a dire emergency and this is a life-threatening condition. The treatment is initially conservative but may require surgical intervention and formation of stoma if the diarrhea is uncontrolled by conservative treatment. Bowel wash-outs are an integral part of treatment of enterocolitis.
  **d. Constipation:** Neonate presenting with constipation and who is stable and feeding appropriately should be investigated before treating.
4. The following investigations are to be done in a suspected case of Hirschsprung's disease:
  - A plain AP view erect abdominal X-Ray shows multiple loops of dilated bowel, absence of rectal gas shadow, loaded colon, and multiple air-fluid levels (with intestinal obstruction) and gas under diaphragm in perforative peritonitis.
  - Barium/Contrast enema: about 70-75% of HD can be diagnosed with barium or contrast enemas. The features diagnostic of HD are transitional zone, absence peristalsis in aganglionic segment, saw-tooth appearance in aganglionic segment, retention of barium into the colon after 24 hours. Total colonic aganglionosis can be missed if the entire colon and distal ileum is not included in this radiological study. Also, the classical radiologic features are absent in about 25% of neonates. [3]
  - Anal manometry is not conclusive in neonates and should not be a primary diagnostic modality. If necessary if should be done only after 2 weeks after birth as maturity of reflex is a pre-requisite for reliable reports.
  - Rectal biopsy is the gold standard for diagnosis of HD. Rectal biopsy could be done either as a suction rectal biopsy, which is an OPD procedure, or as full-thickness rectal biopsy (FTRB). The facilities for study of acetyl-cholinesterase must be available if suction rectal biopsy is undertaken.
  - Mutliple seromuscular biopsies: This is done only if child is explored for complications as mentioned above and is usually the first stage of the staged repair of HD.
5. Surgical intervention is mandatory in all patients with HD. The timing of surgery and the method of surgical correction is based on type of HD, condition of the child and the expertise available.
The various surgical options are:
  - Single staged – Primary pull-through. Pre-requisite for this is a non-dilated bowel, which could be achieved by regular and frequent bowel washouts.
  - Two-staged surgery, where end or loop colostomy is done at the transitional level in the first stage, followed by pull-through of the stoma in the second stage. Pre-requisite is availability of frozen section. The siting of end or loop colostomy is done based on frozen section report of presence of ganglion cells.
  - 3 staged procedure – where transvers colostomy and multiple seromuscular biopsies are followed by pull-through and then colostomy closure. This has been traditionally the most often chosen option and is now no longer in vogue due to availability of newer techniques.
The various options for pull-through available are:
  - Duhamel’s recto-anal pull-through
  - Soave’s endoanal pull-through
  - Swenson’s pull-through
  The surgical intervention could be done either open or laparoscopically.
6. Post-surgery majority of the children with Hirschsprung’s disease improve dramatically, but the following are the long-term issues causing morbidity in patients:
  **1. Enterocolitis: ** The incidence of post-operative enterocolitis is much less than that in the pre-operative phase. About 35 percent of children have at least one episode of enterocolitis after surgery and some children have recurrent or prolonged episodes of enterocolitis. IV antibiotics especially Metronidazole and rectal irrigation are the mainstays of treatment.
  **2. Incontinence: **This occurs usually in cases where the anastomosis is done below the dentate line or when there is damage to the sphincter during the pull-through surgery.
  **3. Constipation: **This occurs mainly because of residual or secondary aganglionosis, but sometimes even in those who do have normal pulled-through bowel. Further surgical intervention following diagnosis of residual aganglionosis is mandatory.
7. Majority of neonates (about 70%) with HD have an isolated lesion. Nevertheless, well documented associated anomalies are as follows [4-6]:
  - Down syndrome : in ~ 5- 10% of HD cases
  - Congenital Deafness : 9%
  - Congenital heart defects : 8%
  - Mental reatardation and seizures : 6%
  - Neurocristopathy syndromes
    - Waardenburg-Shah syndrome
    - Haddad syndrome
    - MEN IIa
  - other non-neurocristopathy syndromes
    - Aarskog syndrome
    - Bardet-Biedl syndrome
    - Fryns syndrome
    - Pallister-Hall syndrome
    - Smith-Lemli-Opitz syndrome
    - Congenital central hypoventilation syndrome

## Footnotes

**Source of Support:** Nil

**Conflict of Interest:** None

